# Supplementary material for: Mitochondrial dysfunction abrogates dietary lipid processing in enterocytes
Source: Nature. 2023 Dec 20;625(7994):385–92. doi: 10.1038/s41586-023-06857-0 (PMC10781618; doi:10.1038/s41586-023-06857-0)
Supplement: Supplementary file 1 — This file contains Supplementary Fig. 1 and Supplementary Tables 4 and 5. Supplementary Fig. 1: Uncropped gels from immunoblots presented in the manuscript. Supplementary Table 4: List of the genotypes and the numbers of mice for which histological sections were analysed for each experimental group. Supplementary Table 5: SYBR Green primer sequences used for RT–qPCR. [file 41586_2023_6857_MOESM1_ESM.pdf]

nature portfolio

https://doi.org/10.1038/s41586-023-06857-0

Supplementary information

Mitochondrial dysfunction abrogates dietary lipid processing in enterocytes

In the format provided by the authors and unedited

Supplementary Figure 1

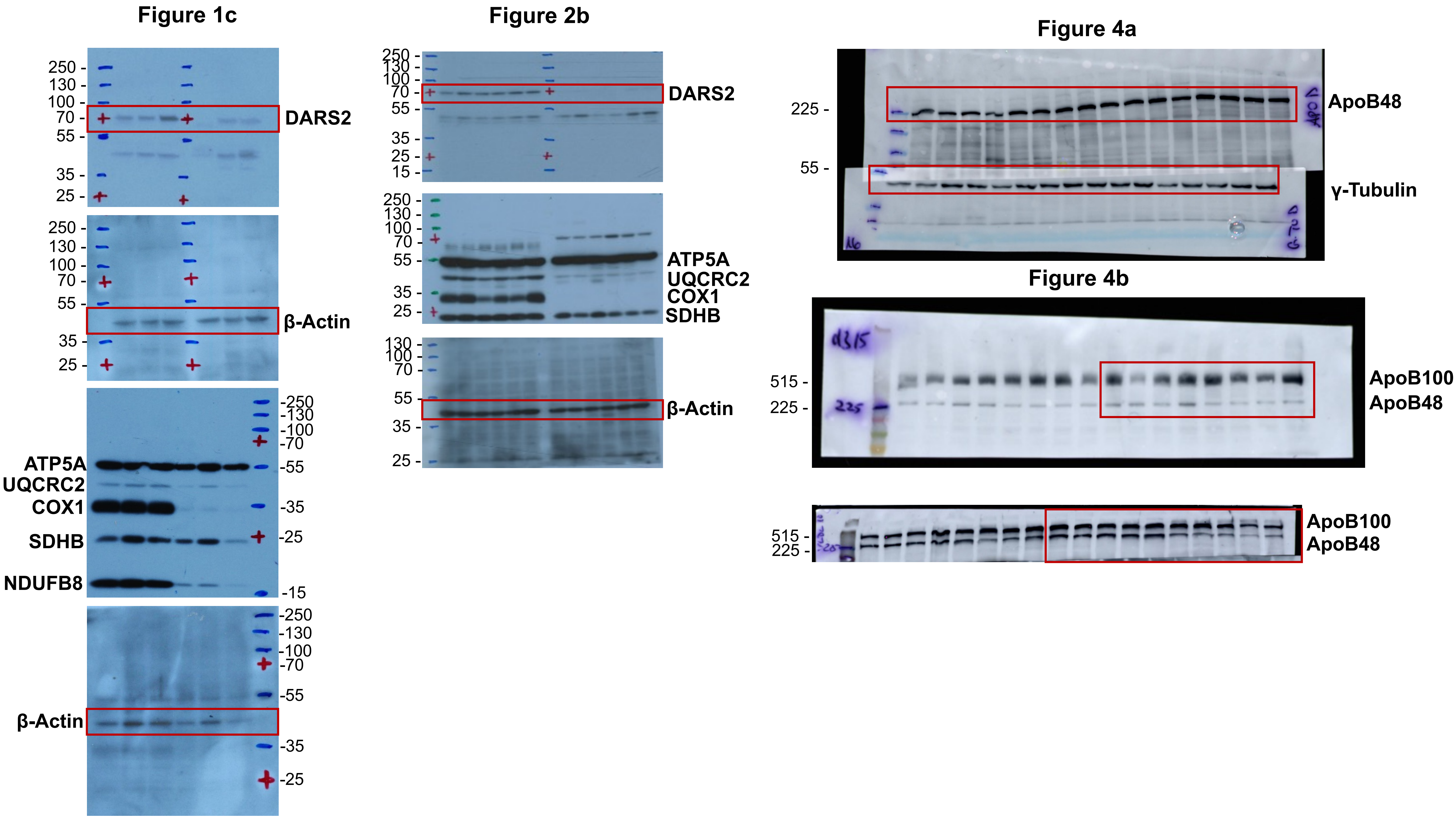

Supplementary Figure 1 (continued)

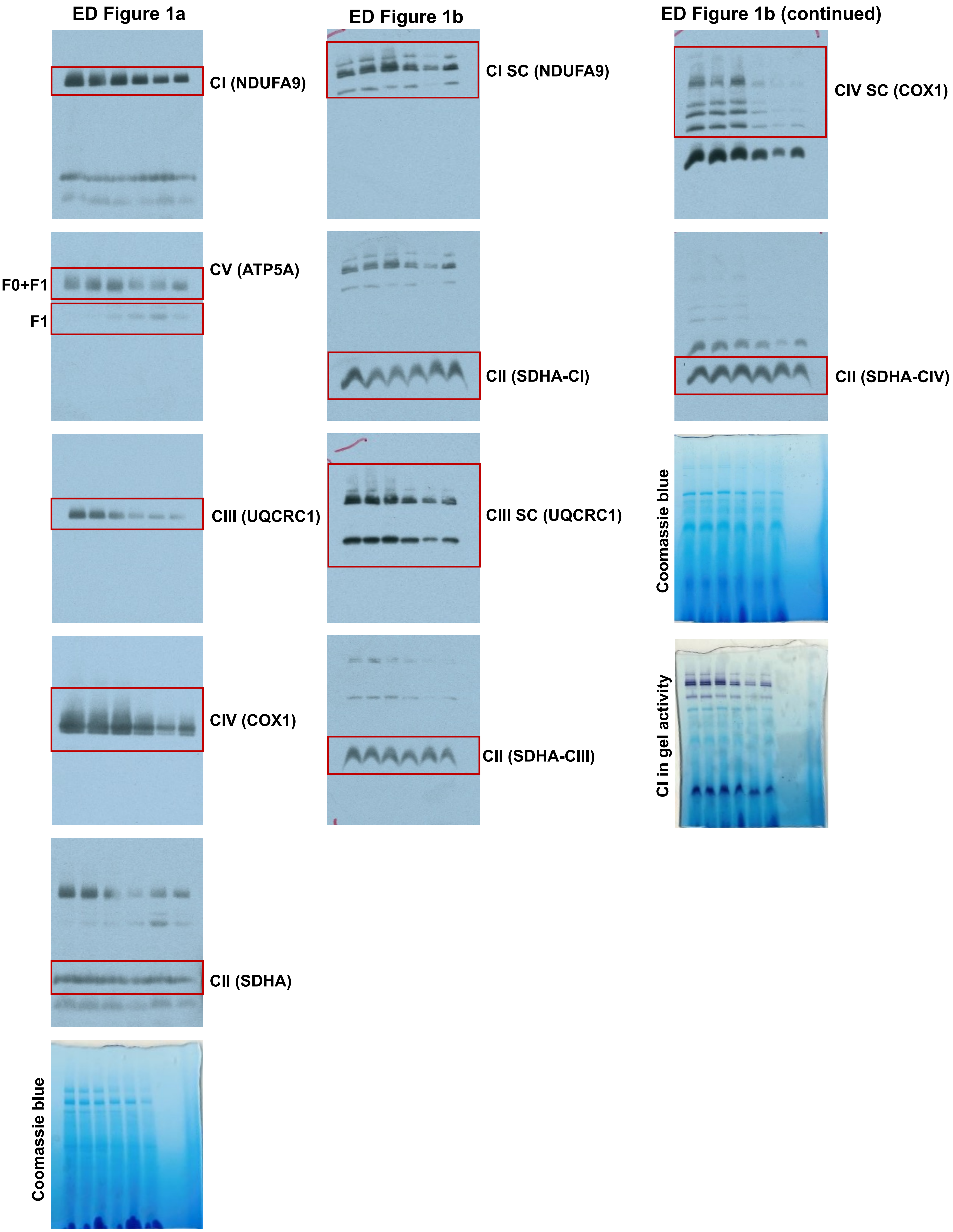

Supplementary Figure 1 (continued)

ED Figure 3e

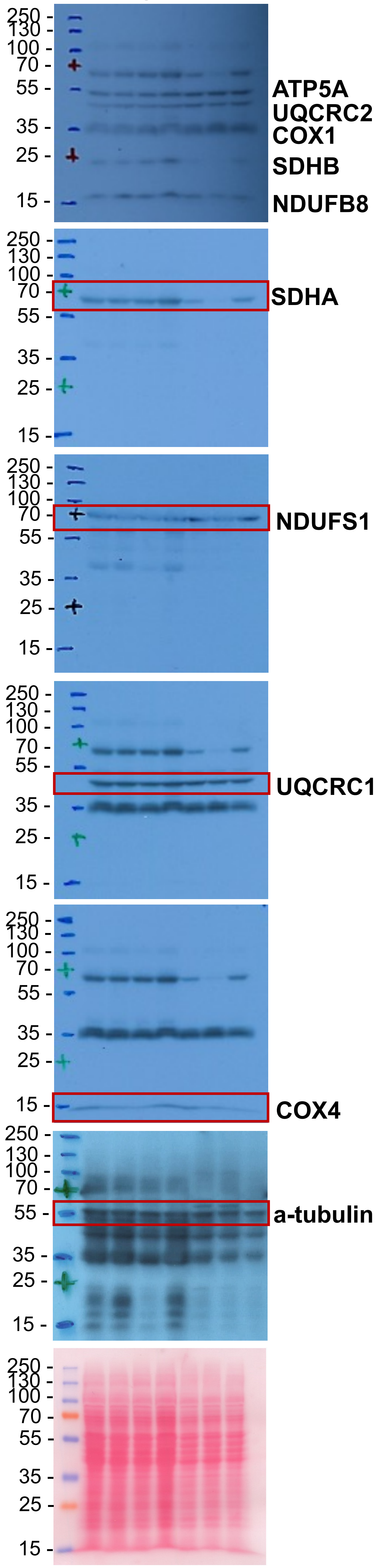

ED Figure 3j

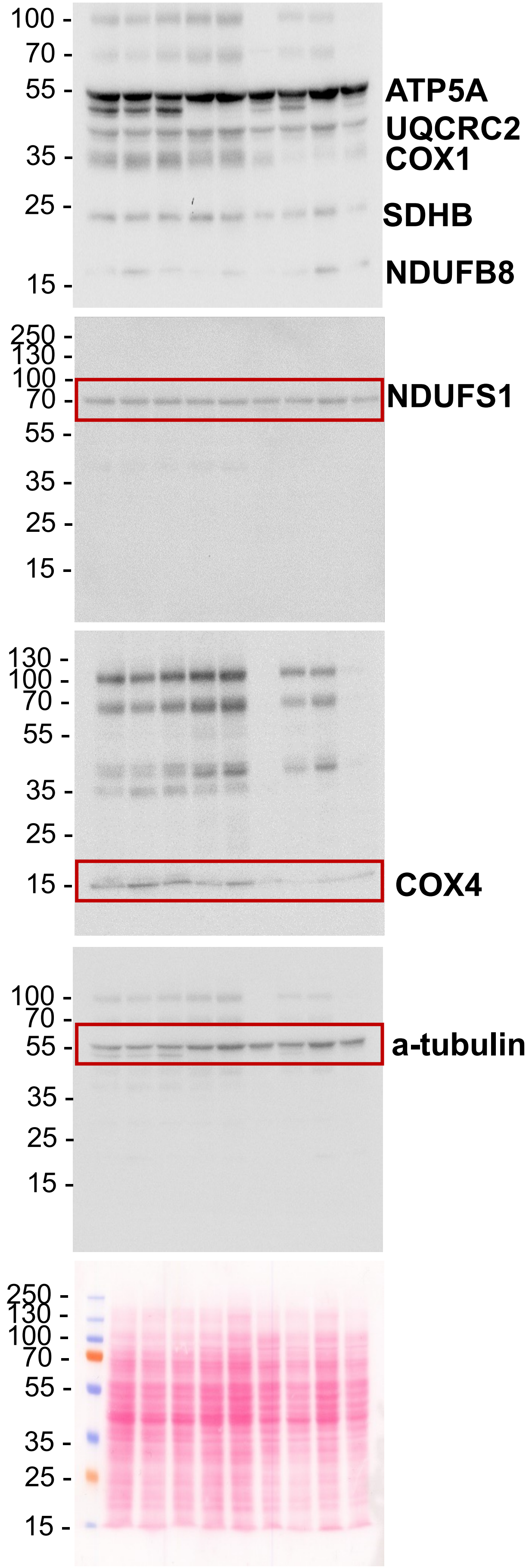

ED Figure 4c

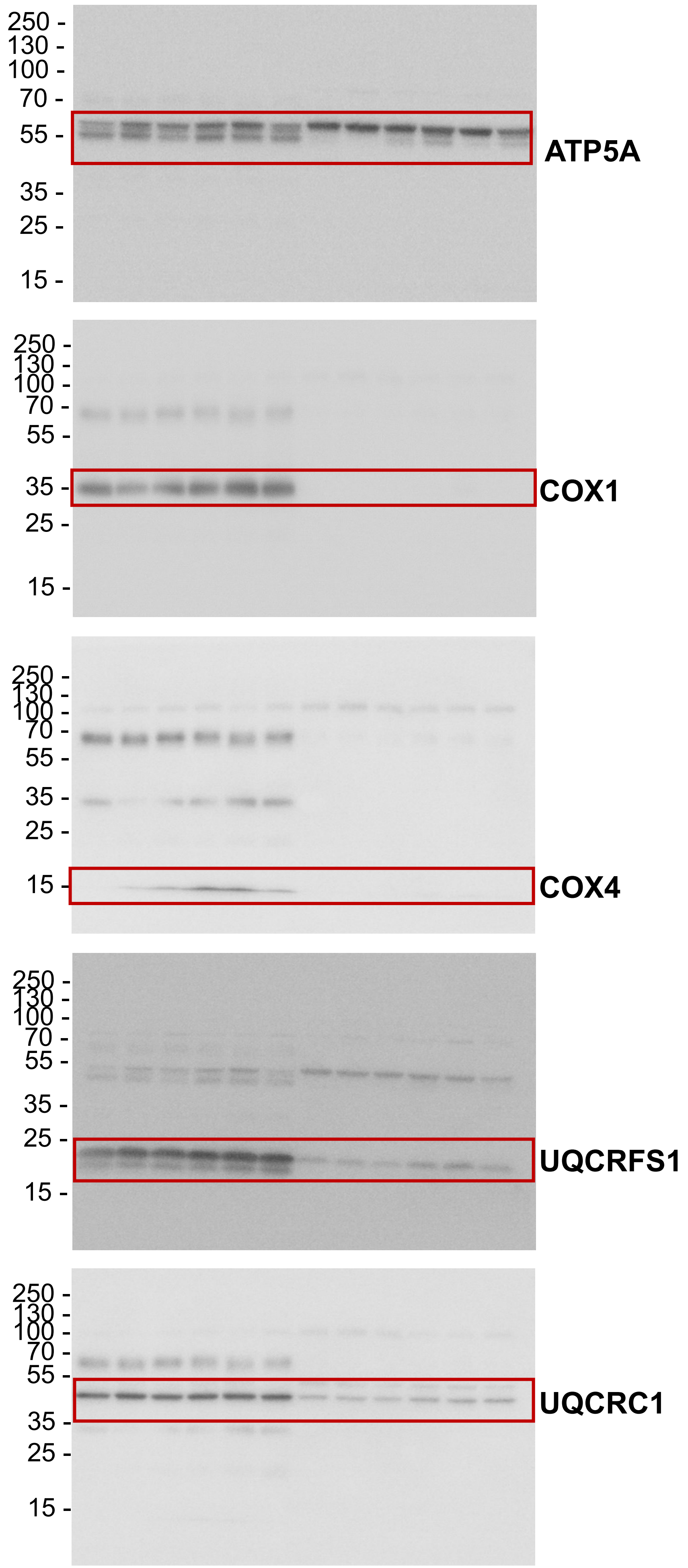

Supplementary Figure 1 (continued)

ED Figure 4c (continued)

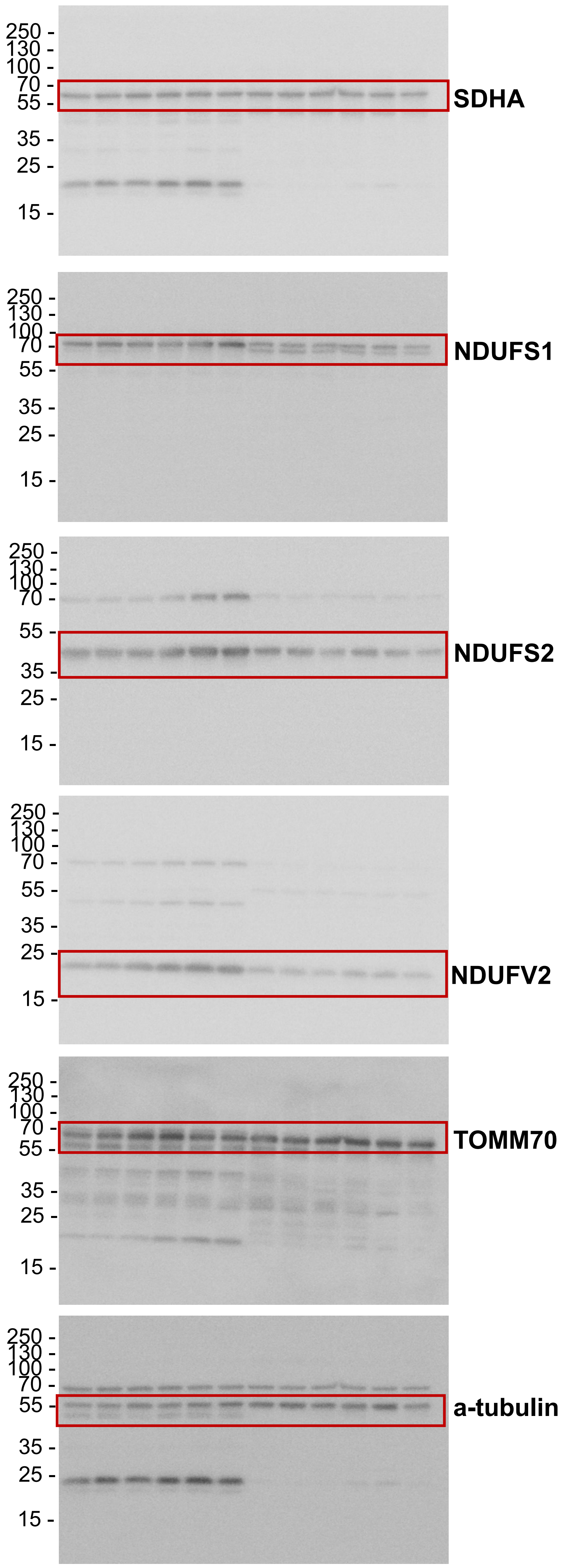

ED Figure 5b

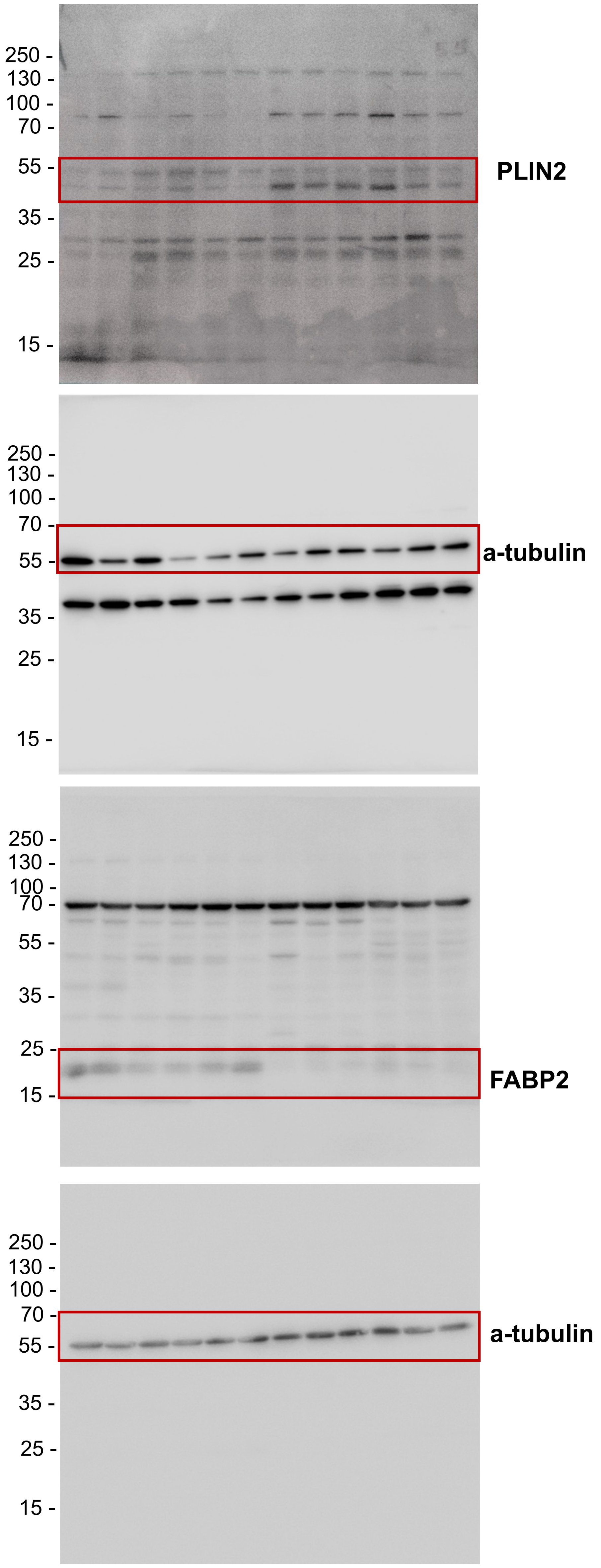

ED Figure 5b (continued)

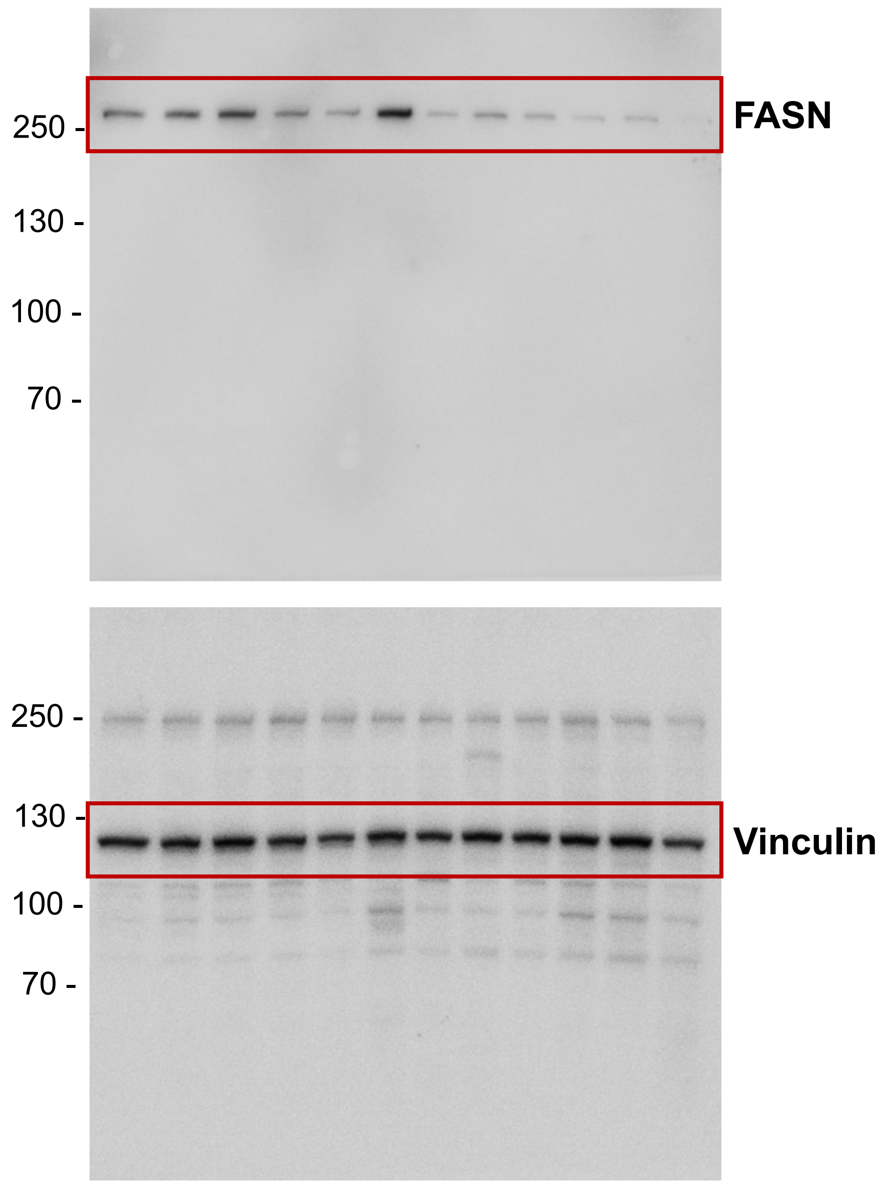

Supplementary Figure 1 (continued)

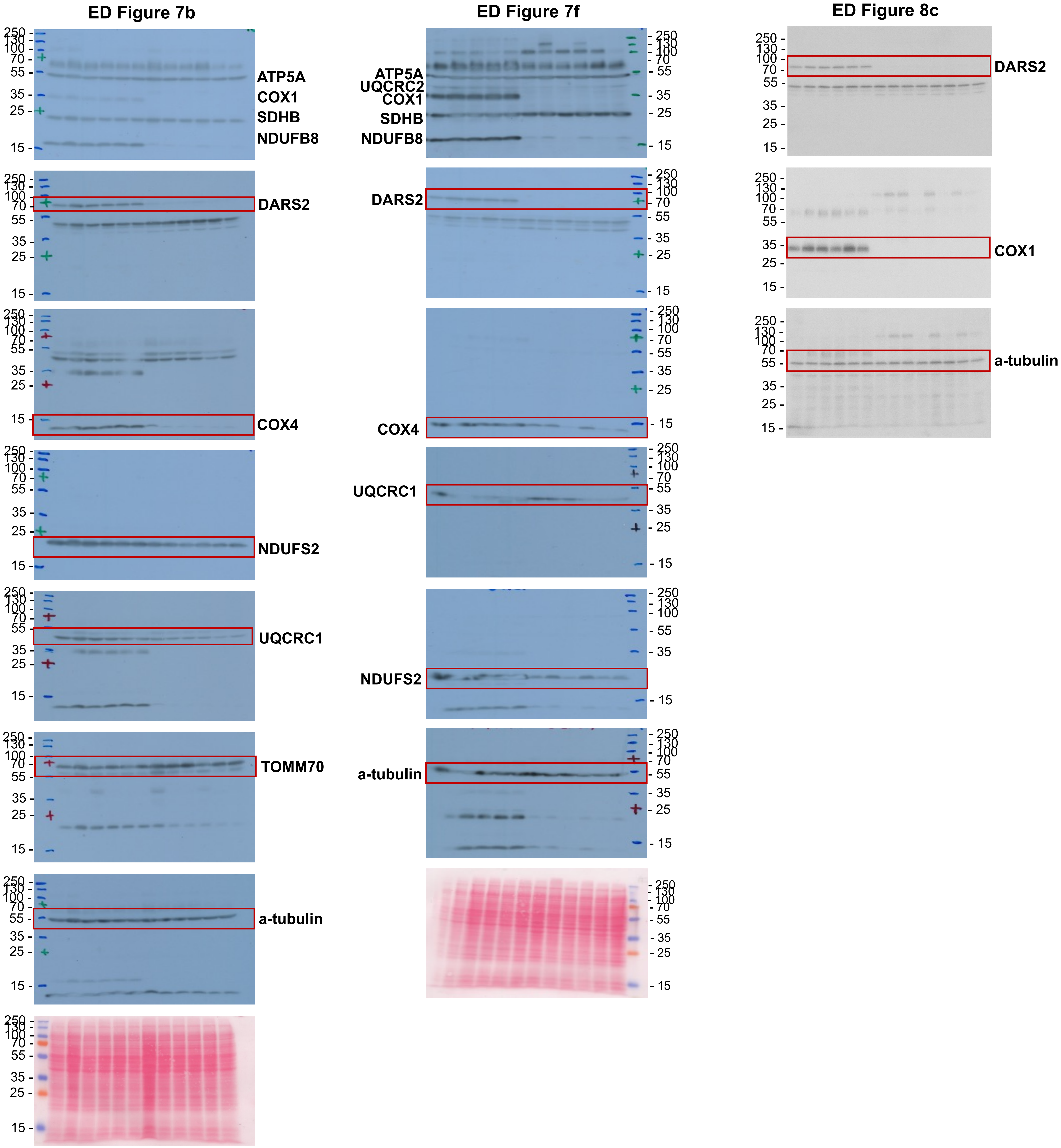

Supplementary Figure 1. Uncropped gels from immunoblots presented in the manuscript.

Genotype	Age	Whole Small intestine												
		Stainings												
		H/E	COX/SDH	Ki67	PLIN2	ORO	ALP	PAS	OLFM4	CC3	CC8	CD45	F4/80	
$Dars2^{fl/fl}$ or *$Dars2^{fl/wt}$*	6-7 days-old	17	8	6	5	12	6	6	9	9	9	8	6	**Figure 1d,1f & Ext. Figure 1d,1e**
$DARS2^{IEC-KO}$		19	8	6	5	12	6	6	9	9	9	8	6	

Genotype	Age	H/E	COX/SDH	Ki67	PLIN2	ORO	
$Sdha^{fl/fl}$ or *$Sdha^{fl/wt}$*	6-7 days-old	10	5	5	5	8	**Ext. Figure 3d**
$SDHA^{IEC-KO}$		11	5	5	5	8	
$Cox10^{fl/fl}$ or *$Cox10^{fl/wt}$*	6-7 days-old	7	5	5	5	5	**Ext. Figure 3i**
$COX10^{IEC-KO}$		8	5	5	5	5	

Genotype	Days after last tamoxifen injection	Proximal Small intestine									
		Stainings									
		H/E	COX/SDH	Ki67	PLIN2	ORO	ALP	PAS	CC3	CD45	
$Dars2^{fl/fl}$	7d, 8d NCD	31	6	7	5	12	7	7	7	7	**Figure 2d, 2e, Figure 3a (ORO), Ext. Figure 6b**
$DARS2^{tamIEC-KO}$		31	6	7	5	12	7	7	7	7	

Genotype	Days after last tamoxifen injection	H/E	COX/SDH	Ki67	PLIN2	ORO	
$Dars2^{fl/fl}$	3d NCD	6	6	6	6	7	**Ext. Figure 7d**
$DARS2^{tamIEC-KO}$		6	6	6	6	7	
$Dars2^{fl/fl}$	5d NCD	10	6	6	6	7	**Ext. Figure 7h**
$DARS2^{tamIEC-KO}$		11	6	6	6	7	
$Dars2^{fl/fl}$	7d FFD	32	6	7	7	12	**Figure 3a**
$DARS2^{tamIEC-KO}$		32	6	7	7	12	

Genotype	Days after last tamoxifen injection	Distal Small intestine									
		Stainings									
		H/E	COX/SDH	Ki67	PLIN2	ORO	ALP	PAS	CC3	CD45	
$Dars2^{fl/fl}$	7d, 8d NCD	31	12	7	6	12	7	7	7	7	**Figure 2e, Ext. Figure 6c, 6d**
$DARS2^{tamIEC-KO}$		31	12	7	6	12	7	7	7	7	

Genotype	Days after last tamoxifen injection	H/E	COX/SDH	Ki67	PLIN2	ORO	
$Dars2^{fl/fl}$	7d FFD	32	6	7	7	12	**Ext. Figure 8d**
$DARS2^{tamIEC-KO}$		32	6	7	7	12	

Genotype	Days after last tamoxifen injection	Proximal Small intestine		
		Stainings (IF)		
		TGN38/E-cadherin/PLIN2/DAPI	TGN38/E-cadherin/DAPI	
$Dars2^{fl/fl}$	7d, 8d NCD	12	6	**Ext. Figure 10a, 10b, 10c**
$DARS2^{tamIEC-KO}$		12	6	
$Dars2^{fl/fl}$	3d NCD	6	6	**Ext. Figure 10a, 10b**
$DARS2^{tamIEC-KO}$		6	6	
$Dars2^{fl/fl}$	5d NCD	6	6	**Figure 4d, Ext. Figure 10a**
$DARS2^{tamIEC-KO}$		6	6	
$Dars2^{fl/fl}$	7d FFD	6	**Ext. Figure 10c**	
$DARS2^{tamIEC-KO}$		6		

Genotype	Days after last tamoxifen	Distal Small intestine	
		Stainings (IF)	
		TGN38/E-cadherin/PLIN2/DAPI	
$Dars2^{fl/fl}$	7d, 8d NCD	6	**Ext. Figure 10b**
$DARS2^{tamIEC-KO}$		6	

Supplementary Table 4. List of the genotypes and the numbers of mice for which histological sections were analyzed for each experimental group.

Name	Forward (5´- 3´)	Reverse (5´- 3´)
Ppara	AACATCGAGTGTCGAATATGTGG	CCGAATAGTTCGCCGAAAGAA
Scd1	GCTCTACACCTGCCTCTTC	CCGTGCTTGTAAGTTCTG
Acc1	GACAGAGGAAGATGGCGTCC	TACAACTTCTGCTCGCTGGG
Srepb1c	CGCGGAAGCTGTCGGGGTAG	AAATGTGCAATCCATGGCTCCGTGGTC
Dgat1	GCCCCATGCGTGATTATT	TCTGTCAGGGCACCCACT
Acox1	TAACTTCCTCACTCGAAGCCA	AGTTCCATGACCCATCTCTGTC
Mgat2	GTGTGGGATTAGGGGGACTT	TCCCTGTTTGTCCTTTGGTC
Mcad	AGGGTTTAGTTTTGAGTTGACGG	CCCCGCTTTTGTCATATTCCG
Ehhadh	ATGGCTGAGTATCTGAGGCTG	GGTCCAAACTAGCTTTCTGGAG
Fasn	GGAGGTGGTGATAGCCGGTAT	TGGGTAATCCATAGAGCCCAG
Pparg	CCTGAAGCTCCAAGAATACCA	GCCTGATGCTTTATCCCCACA
Mttp	TGAGCGGCTATACAAGCTCAC	CTGGAAGATGCTCTTCTCGC
Lipe	AGTGCCTATTCAGGGACAGA	TGGGCGATGTGGTCTTTT
Mgat1	CTGGTTCTGTTTCCCGTTGT	TGGGTCAAGGCCATCTTAAC
Gpat3	GTGCTGGGTGTCCTAGTGC	AAGCTGATCCCAATGAAAGC
Acly	CAGTCCCAAGTCCAAGATCC	GTCTCGGGAGCAGACATAGT
Hadha	TGCATTTGCCGCAGCTTTAC	GTTGGCCCAGATTTCGTTCA
Acadvl	CTACTGTGCTTCAGGGACAAC	CAAAGGACTTCGATTCTGCCC
Cd36	CTGTGGGCTCATTGCTGG	CGCCACGTCATCTGGGTTT
Cpt2	CAGCACAGCATCGTACCCA	TCCCAATGCCGTTCTCAAAAT
Atgl	GGATGGCGGCATTTCAGACA	CAAAGGGTTGGGTTGGTTCAG
Lpl	ACTCGCTCTCAGATGCCCTA	TTGTGTTGCTTGCCATTCTC
Hprt1	GCCCCAAAATGGTTAAGGTT	TTGCGCTCATCTTAGGCTTT

Supplementary Table 5. SYBR Green primer sequences used for quantitative RT-PCR.
