# Supplementary material for: Mitochondrial dysfunction abrogates dietary lipid processing in enterocytes
Source: Nature. 2023 Dec 20;625(7994):385–92. doi: 10.1038/s41586-023-06857-0 (PMC10781618; doi:10.1038/s41586-023-06857-0)
Supplement: Supplementary file 2 — Reporting Summary [file 41586_2023_6857_MOESM2_ESM.pdf]

nature research

Corresponding author(s): Manolis Pasparakis

Last updated by author(s): Aug 29, 2023

Reporting Summary

Nature Research wishes to improve the reproducibility of the work that we publish. This form provides structure for consistency and transparency in reporting. For further information on Nature Research policies, see Authors & Referees and the Editorial Policy Checklist.

Statistics

For all statistical analyses, confirm that the following items are present in the figure legend, table legend, main text, or Methods section.

n/a	Confirmed	
☐	☒	The exact sample size (*n*) for each experimental group/condition, given as a discrete number and unit of measurement
☐	☒	A statement on whether measurements were taken from distinct samples or whether the same sample was measured repeatedly
☐	☒	The statistical test(s) used AND whether they are one- or two-sided<br>*Only common tests should be described solely by name; describe more complex techniques in the Methods section.*
☒	☐	A description of all covariates tested
☐	☒	A description of any assumptions or corrections, such as tests of normality and adjustment for multiple comparisons
☐	☒	A full description of the statistical parameters including central tendency (e.g. means) or other basic estimates (e.g. regression coefficient) AND variation (e.g. standard deviation) or associated estimates of uncertainty (e.g. confidence intervals)
☐	☒	For null hypothesis testing, the test statistic (e.g. *F*, *t*, *r*) with confidence intervals, effect sizes, degrees of freedom and *P* value noted<br>*Give P values as exact values whenever suitable.*
☒	☐	For Bayesian analysis, information on the choice of priors and Markov chain Monte Carlo settings
☒	☐	For hierarchical and complex designs, identification of the appropriate level for tests and full reporting of outcomes
☒	☐	Estimates of effect sizes (e.g. Cohen's *d*, Pearson's *r*), indicating how they were calculated

Our web collection on statistics for biologists contains articles on many of the points above.

Software and code

Policy information about availability of computer code

Data collection	Histological images were collected by using a light microscope equipped with a KY-F75U digital camera (JVC) (DM4000B, Leica Microsystems, Diskus 4.50 software).<br>Immunofluorescence images were acquired using a TCS SP8 confocal laser scanning microscope (Inverse, DMi 8 CS, Leica Microsystems LAS X, Lightning software version 5.1.0), a LSM Meta 710 confocal laser scanning microscope (Carl Zeiss Technology, ZEN 2009 software), a Spinning Disc Confocal Microscope (Inverse, Nikon TiE, UltraView VoX, Perkin Elmer, Volocity software) and an optical Zeiss Axio Imager Z1 microscope (ZEN 2009 software).<br>Electron microscopy images were captured by a transmission electron microscope (JOEL JEM2100 Plus) at an acceleration voltage of 80 kV, using a 4K-CCD camera, OneView (GATAN).<br>The rt-PCR data was collected by QuantStudio 12K Flex Software v1.6 (Applied Biosystems).<br>For RNA sequencing analysis, gene expression was determined using QuantSeq 3' mRNA-Seq Library Prep Kit FWD for Illumina (Lexogen). QuantSeq libraries were sequenced on an Illumina NovaSeq 6000 sequencer using Illumina RTA v3.4.4 base-calling software.<br>Western blot data was collected by Curix 60 Processor and western blot imager (FUSION Solo X, Vilber).<br>Serum data was collected by Biochemical Cobas C111 Analyzer (Roche Diagnostics).<br>Plasma data was collected by fast performance liquid chromatography (FPLC) on a Superose 6 10/300 GL column (GE Healthcare). For the isolation of the TRL fractions, ultracentrifugation was performed using a Beckman Optima MAX-XP in a Beckman TL100 rotor.<br>Metabolic tracing data was collected by Perkin Elmer Tricarb Scintillation Counter.<br>Lipidomics data was collected by using QTRAP 6500 system coupled to a 1260 Infinity Binary LC (Agilent) or to a Nexera X2 UHPLC (Shimadzu) and TriVersa NanoMate (Advion).<br>Metabolomics data was collected by Thermo Scientific Q Exactive Hybrid Quadrupole-Orbitrap Mass spectrometer (HRMS) coupled to a Dionex Ultimate 3000 UHPLC, Orbitrap Eclipse™ Tribrid™ Mass Spectrometer (Thermo Fisher Scientific).<br>Proteomics data was collected by liquid chromatography tandem mass spectrometry on an Orbitrap Eclipse™ Tribrid™ Mass Spectrometer (Thermo Fisher) with FAIMS Pro device.
Data analysis	Image processing and analysis was performed using Photoshop (version 22.4.2 and 23.5.1) and the open-source software FIJI/ImageJ (version 1.53c and 2.0.0.-rc-46/1.50g).

For analysis of the RNA sequencing data, reads were mapped to a mouse reference genome Mus_musculus.GRCm38.100.gtf which was downloaded from (ftp://ftp.ensembl.org/pub/release-100/gtf/mus_musculus/), augmented by entries for the ERCC92 Spike Ins downloaded from (https://assets.thermofisher.com/TFS-Assets/LSG/manuals/ERCC92.zip) and using subread-align (v2.0.1). Illumina adapters were clipped off the raw reads using Cutadapt. All analyses were done in R-4.0.0, using functionality of Bioconductor (v.3.11).
Western blot data produced by FUSION Solo X (Vilber) were visualized using GIMP (v.2.10.20).
Proteomics data analysis was performed by using MaxQuant analysis software and the implemented Andromeda software (1.6.14). Peptides and proteins were identified using the canonical mouse UniProt database (downloaded 08/2019) and statistical analysis was performed using Perseus (1.5.8.5) software. Identified proteins were annotated with the following Gene Ontology terms: Biological Process, Molecular Function, and Cellular Compartment, and the Reactome Pathway database.
Analysis of the metabolomics data was performed by using the Thermo Fisher software Tracefinder (v.5.0), the muma package (v.1.4), the R package „gtools"(v.3.8.2) (https://cran.r-project.org/web/packages/gtools/index.html), and the R base package stats (v.4.0.5) (https://www.r-project.org/). Lipidomics data was analyzed by TraceFinder, Compound Discoverer (Thermo Scientific), MultiQuant, LipidView, MarkerView, PeakView, MasterView (SCIEX).
Gene set enrichment analysis (GSEA) was performed by using gene sets published on the MsigDB (Reactome, KEGG, Biocarta and Hallmarks) (Subramanian, A. et al., 2005) and by (Han, J. et al., 2013) using the packages fgsea (v.1.16.0) (Sergushichev, A. A. et al., 2016) and GSEABase (v.1.52.1) (Morgan, S. et al., 2021). The over representation analysis (ORA) was performed using the clusterProfiler package 22 (v.3.16.1). Volcano plots were generated using the EnhancedVolcano package (v.1.8.0). Graphical visualization was additionally achieved using Instant Clue software (v.0.5.3) (Nolte, H., MacVicar, T. D., et al., 2018).
The output data are plotted using the "emapplot" function of the enrichplot package (v.1.8.1) (https://www.bioconductor.org/packages/release/bioc/html/enrichplot.html).
Detailed code, data tables and figures are available under:
https://github.com/ChristinaSchmidt1/Mitochondria_in_Intestinal_Lipid-transport.
Data compiling and processing was performed using Microsoft Excel 2021 (v.2108) and Adobe Illustrator (v.26.5).
Statistical analysis was performed with GraphPad Prism 6 (v.6.01) and 9 (v.9.4.1).
Schematics were created with BioRender.com.

For manuscripts utilizing custom algorithms or software that are central to the research but not yet described in published literature, software must be made available to editors/reviewers. We strongly encourage code deposition in a community repository (e.g. GitHub). See the Nature Research guidelines for submitting code & software for further information.

Data

Policy information about availability of data

All manuscripts must include a data availability statement. This statement should provide the following information, where applicable:

- Accession codes, unique identifiers, or web links for publicly available datasets
- A list of figures that have associated raw data
- A description of any restrictions on data availability

The original RNA sequencing data are uploaded and available in NCBI's Gene Expression Omnibus and are accessible through GEO Series accession number GSE207803 (https://www.ncbi.nlm.nih.gov/geo/query/acc.cgi?acc=GSE207803).
The original proteomic data are uploaded and available at the DATABASE under accession PXD026934. The mass spectrometry proteomics data have been deposited to the ProteomeXchange Consortium via the PRIDE partner repository with the dataset identifier PXD026934.
The original metabolomics data haven been deposited at Metabolomics Workbench (https://www.metabolomicsworkbench.org) under the study_id ST002184 (datatrack_id:3289, http://dx.doi.org/10.21228/M8X99S).
Numerical source data giving rise to graphical representations and statistical descriptions presented in the Figs. 1–4 and Extended Data Figs. 1, 2, 3, 5, 6, 7, 8, 9 and 11 are provided as Source Data files with the paper.
Uncropped images of immunoblots presented in the figures are included in Supplementary Figure 1.

Field-specific reporting

Please select the one below that is the best fit for your research. If you are not sure, read the appropriate sections before making your selection.

☒ Life sciences ☐ Behavioural & social sciences ☐ Ecological, evolutionary & environmental sciences

For a reference copy of the document with all sections, see nature.com/documents/nr-reporting-summary-flat.pdf

Life sciences study design

All studies must disclose on these points even when the disclosure is negative.

Sample size	Sample size was determined empirically and was based on our previous mouse work. We aimed for a number of at least 3 animals per group to allow basic statistical analysis while using a justifiable number of mutant mice. All sample sizes were annotated within the respective Figure legends. Based on previous experience from similar studies, in vitro experiments with cultured cells were performed at least 3 times (3 biological replicates) to confirm reproducibility. Each biological replicate is defined as an independent culture of cells.
Data exclusions	No animal data was excluded from the analyses.<br>Sample exclusion criteria (OD260/280 <1.8, OD260/230 <1.5 and RIN<7) were applied for QuantSeq 3' mRNA sequencing analysis. Genes were excluded from a DESeq2 run if they had a zero count.<br>For metabolomic analysis, the peak area for each detected metabolite was subjected to the "Filtering 80% Rule", half minimum missing value imputation, and normalized against the total ion count (TIC) of that sample to correct any variations introduced from sample handling through instrument analysis. Samples were excluded after performing testing for outliers based on geometric distances of each point in the PCA score

	plot. For proteomic analysis, potential contaminants and reverse peptides were excluded.
Replication	For in vivo studies we analyzed at least 6 mice per genotype to ensure the reproducibility of the results. For in vitro studies we independently replicated all experiments at least 3 times.<br>For C. elegans studies, at least 2 independent technical replicates were used. All attempts of replication were successful and results were reliably reproduced with the same trend.
Randomization	No specific method of randomization had been used to select animals. We compared groups of mice with different genotypes to assess the effect of specific genetic mutations in the phenotype. Group allocation was thus determined by the genotype of the mice.<br>For C. elegans experiments, worms were randomly selected and were not allocated into groups.
Blinding	No blinding was done during the group generation as the group allocation was determined by the genotype of the mice.<br>For 1-week-old mice, sample collection was not performed in a blinded manner as they exhibited an obvious macroscopic phenotype that revealed the sample identity.<br>Histological evaluation of intestinal sections was performed blindly.<br>Golgi quantification was performed by manually classifying the TGN38 pattern in each cell in one of the five Golgi phenotypes by the same observer, who was blind to the experimental conditions.<br>Metabolomic samples were analysed with LC–MS in a blinded manner.

Reporting for specific materials, systems and methods

We require information from authors about some types of materials, experimental systems and methods used in many studies. Here, indicate whether each material, system or method listed is relevant to your study. If you are not sure if a list item applies to your research, read the appropriate section before selecting a response.

Materials & experimental systems

n/a	Involved in the study	
☐	☒	Antibodies
☐	☒	Eukaryotic cell lines
☒	☐	Palaeontology
☐	☒	Animals and other organisms
☒	☐	Human research participants
☒	☐	Clinical data

Methods

n/a	Involved in the study	
☒	☐	ChIP-seq
☒	☐	Flow cytometry
☒	☐	MRI-based neuroimaging

Antibodies

Antibodies used

Primary antibodies:

1) Monoclonal rabbit anti-Olfm4 (D6Y5A) XP®, Cell Signaling, Cat. No. 39141, Clone name: D6X5A, Lot. No. 1
2) Polyclonal guinea-pig anti-adipophilin / perilipin 2 (N-terminus aa 1-16), Progen, Cat. No. GP46, Lot No. 05/09(0270)NT-02
3) Monoclonal rat anti-Ki67, DAKO, Cat. No. M724901, Clone name: 1O15, Lot No. TEC-3
4) Rabbit polyclonal anti-cleaved Caspase 3, Cell Signaling Technology, Cat. No. 9661, Lot. No. 433
5) Rabbit monoclonal anti-cleaved Caspase 8 (Asp387), Cell Signaling Technology, Cat. No. 8592, Clone name: D5B2, Lot. No. 3
6) Monoclonal rat anti-F4/80, AbD Serotec, Cat. No. MCA497, Clone name: A3-1
7) Monoclonal rat anti-CD45, BD Biosciences, Cat. No. 560510, Clone name: 30-F11, Lot. No. E03735-1631
8) Polyclonal sheep anti-TGN38, bio-techne, Cat. No. AF8059-SP, Lot. No CHWO012104A
9) Purified mouse anti-E-cadherin, BD Biosciences, Cat. No. 610182, Clone name: 36/ E-Cadherin (RUO), Lot. No 9315423
10) Polyclonal rabbit anti-DARS2, Proteintech, Cat. No. 13807-1-AP, Lot. No 00019410
11) Total OXPHOS Rodent WB Antibody Cocktail, Abcam, Cat. No. ab110413, Lot. No K2342
12) Polyclonal goat anti-β-actin, Santa Cruz, Cat. No. sc-1616, Clone number: I-19, Lot. No B1116
13) Mouse monoclonal anti-MTCO1/COX1, Molecular Probes, Cat. No 459600, Clone name: 1D6E1A8, Part/Lot. No. 459600/G1010
14) Mouse monoclonal anti-COX4L1, Molecular Probes, Cat. No A21348, Clone name: 20E8C12, Lot. No. 1801178
15) Mouse monoclonal anti-UQCRC1, Molecular Probes, Cat. No 459140, Clone name: 16D10AD9AH5, Part/Lot. No. 459140/IC3878
16) Rabbit polyclonal anti-NDUFS1, Proteintech, Cat. No 12444-1-AP, Lot. No. 00007233
17) Rabbit polyclonal anti-NDUFS2, Abcam, Cat. No ab96160, Lot. No. GR3179687-4
18) Rabbit polyclonal anti-NDUFV2, Proteintech, Cat. No 15301-1-AP, Lot. No. 00006404
19) Mouse monoclonal anti-UQCRFS1/RISP, Abcam, Cat. No ab14746, Clone name: 5A5, Lot. No. GR304562-8
20) Mouse monoclonal anti-ATP5A, Abcam, Cat. No ab14748, Clone name: 15H4C4, Lot. No. GR3306993-24
21) Mouse monoclonal anti-SDHA, Molecular Probes, Cat. No 459200, Clone name: 2EGC12FB2AE2, Lot. No. UJ2872542A
22) Mouse monoclonal anti-NDUFA9, Molecular Probes, Cat. No 459100, Clone name: 20C11B11B11, Lot. No. GR3268847-10
23) Mouse monoclonal anti-tubulin, Sigma Aldrich, Cat. No T6074, Clone name: TUBA4A, Lot. No. 046M4763V
24) Rabbit polyclonal anti-TOMM70, Sigma Aldrich, Cat. No. HPA014589, Lot. No. 000010432
25) Rabbit polyclonal anti-FABP2, Proteintech, Cat. No. 21252-1-AP, Lot. No. 00014123
26) Rabbit polyclonal anti-FASN, Cell Signaling, Cat. No. 3189S, Lot. No. 2
27) Rabbit monoclonal anti-Vinculin XP, Cell Signaling Technology, Cat. No. 13901, Clone name: E1E9V, Lot. No. 6
28) Goat Antibody Monospecific for Human Apolipoprotein B, Beckmann Coulter, Cat. No. 467905

Secondary antibodies:

1) Donkey anti-rabbit IgG conjugated to HRP antibody, GE Healthcare, Cat. No. NA934V, Lot. No 17203153
2) Sheep anti-mouse IgG conjugated to HPR antibody, GE Heathcare, Cat. No. NA931, Lot. No 17317435
3) Donkey anti-goat IgG, conjugated to HPR antibody, Jackson Laboratories, Cat. No.705-035-003, Lot. No 17320421
4) Goat anti-guinea Pig IgG conjugated to HPR Antibody, Progen, Cat. No 90001, Lot. No.908211
5) Goat anti-mouse IgG antibody (H+L) Biotinylated, Vector Laboratories, Cat. No. BA-9200, Lot. No. X0623
6) Goat anti-rabbit IgG antibody (H+L) Biotinylated, Vector laboratories, Cat. No. BA-1000, Lot. No. 10180764
7) Goat anti-rat IgG antibody (H+L) Biotinylated, Vector Laboratories, Cat. No. BA-9400, Lot. No. 112632
8) Polyclonal donkey anti-sheep IgG NorthernLights™ NL557-conjugated Antibody, bio-techne, Cat. No. NL010, Lot. No. AADR0713071
9) Polyclonal goat anti-mouse Alexa 488 - fluorescence-conjugated secondary Antibody, Molecular Probes, Cat. No. A1101
10) Polyclonal goat anti-guinea pig Alexa 633 - fluorescence-conjugated secondary Antibody, Molecular Probes, Cat. No. A21105

Validation

The antibodies used in this study were tested by the manufacturer.

Primary antibodies:

1) anti-Olfm4 (D6Y5A) XP® (Cell Signaling, 39141, clone D6X5A) can be found in 83 citations. The manufacturer also provides antibody testing data: https://www.cellsignal.com/products/primary-antibodies/olfm4-d6y5a-xp-rabbit-mab/39141

2) anti- adipophilin/perilipin 2 (N-terminus aa 1-16)(Progen, GP46) can be found in 3 citations. The manufacturer also provides antibody testing data : https://www.progen.com/anti-Perilipin-2-N-terminus-aa-1-16-guinea-pig-polyclonal-serum/GP46

3) anti-Ki67 (DAKO, M724901,1O15) can be found in 2368 citations (https://www.citeab.com/antibodies/2390690-m7240-ki-67-antigen-concentrate)

4) anti-cleaved-caspase3 (Cell Signaling, 9661, Asp175) can be found in 9193 citations. The manufacturer provides antibody testing data and validation: https://www.cellsignal.com/products/primary-antibodies/cleaved-caspase-3-asp175-antibody/9661

5) anti-cleaved-caspase-8 (Cell signaling, 8592, D2B5) can be found in 294 citations. The manufacturer provides antibody testing data and validation: https://www.cellsignal.com/products/primary-antibodies/cleaved-caspase-8-asp387-d5b2-xp-rabbit-mab/8592

6) anti F4/80 (AbD Serotec, MCA497, A3-1) can be found in 230 citations. The manufacturer provides antibody testing data and validation: https://www.bio-rad-antibodies.com/monoclonal/mouse-f4-80-antibody-cl-a3-1-mca497.html?f=purified

7) anti-CD45 (BD Biosciences, 560510,30F11) can be found in 7 citations. The manufacturer provides antibody testing data and validations: https://www.bdbiosciences.com/en-de/products/reagents/flow-cytometry-reagents/research-reagents/single-color-antibodies-ruo/alexa-fluor-700-rat-anti-mouse-cd45.560510

8) anti-TGN38 (bio-techne, AF8059-SP). The manufacturer also provides antibody testing data: https://www.bio-techne.com/p/antibodies/rat-tgn38-antibody_af8059

9) anti-E-cadherin (BD Biosciences, 610182) can be found in 5 citations. The manufacturer also provides antibody testing data: https://www.bdbiosciences.com/en-de/products/reagents/microscopy-imaging-reagents/immunofluorescence-reagents/purified-mouse-anti-e-cadherin.610182

10) anti-DARS-2 (Proteintech, 13807-1-AP) can be found in 4 citations. The manufacturer also provides antibody testing Data and knockout/knockdown validation: https://www.ptglab.com/products/DARS2-Antibody-13807-1-AP.htm#publications

11) Total OXPHOS Antibody Cocktail (Abcam, ab110413) can be found in 1023 citations. The manufacturer also provides antibody testing data: https://www.abcam.com/products/panels/total-oxphos-rodent-wb-antibody-cocktail-ab110413.html

12) anti-ß-actin (Santa Cruz, sc-1616, I-19) can be found in 1651 citations. The manufacturer discontinued the product.

13) anti-MTCO1/COX1 (Molecular probes, 459600, clone 1DE1A8) can be found in 117 citations. The manufacturer also provides antibody test data: https://www.thermofisher.com/antibody/product/MTCO1-Antibody-clone-1D6E1A8-Monoclonal/459600.

14) anti-COX4L1 (Molecular probes, A21348, 20E8C12) can be found in 82 citations. The manufacturer also provides antibody test data: https://www.thermofisher.com/antibody/product/OxPhos-Complex-IV-subunit-IV-Antibody-clone-20E8C12-Monoclonal/A21348

15) anti- UQCRC1 (Molecular probes, 459140, 16D10AD9AH5) can be found in 45 citations. The manufacturer also provides antibody test data: https://www.thermofisher.com/antibody/product/UQCRC1-Antibody-clone-16D10AD9AH5-Monoclonal/459140

16) anti NDUFS1 (Proteintech, 12444-1-AP) can be found in 60 citations. The manufacturer also provides antibody testing data and validation: https://www.ptglab.com/products/NDUFS1-Antibody-12444-1-AP.htm

17) anti-NDUFS2 (Abcam,ab96160) can be found in 7 citations. The manufacturer discontinued the product.

18) anti-NDUFV2 (Proteintech, 15301-1-AP) can be found in 36 citations. The manufacturer also provides antibody testing data and validation: https://www.ptglab.com/products/NDUFV2-Antibody-15301-1-AP.htm

19) anti-UQCRFS1/RISP (Abcam, ab14746) can be found in 77 citations. The manufacturer also provides antibody testing data: https://www.abcam.com/products/primary-antibodies/uqcrfs1risp-antibody-5a5-ab14746.html

20) anti-ATP5A (Abcam, ab14748,15H4C4) can be found in 482 citations. The manufacturer also provides antibody testing data: https://www.abcam.com/products/primary-antibodies/atp5a-antibody-15h4c4-mitochondrial-marker-ab14748.html

21) anti-SDHA (Molecular probes, 459200,2EGC12FB2AE2) can be found in 85 citations. The manufacturer also provides antibody testing data: https://www.thermofisher.com/antibody/product/SDHA-Antibody-clone-2E3GC12FB2AE2-Monoclonal/459200

22) anti-NDUFA9 (Molecular probes, 459100, 20C11B11B11) can be found in 58 citations. The manufacturer also provides antibody testing data: https://www.thermofisher.com/antibody/product/NDUFA9-Antibody-clone-20C11B11B11-Monoclonal/459100

23) anti-Tubulin (Sigma Aldrich, T6074) can be found in 1214 citations. The manufacturer also provides antibody testing data and validations: https://www.sigmaaldrich.com/DE/en/product/sigma/t6074

24) anti-TOMM70 (Sigma Aldrich, HPA014589) can be found in 9 citations. The manufacturer also provides antibody testing data and validation: https://www.sigmaaldrich.com/DE/en/product/sigma/hpa014589

25) anti-FABP2 (Proteintech, 21252-1-AP) can be found in 10 citations. The manufacturer also provides antibody testing data and validations: https://www.ptglab.com/products/FABP2-Antibody-21252-1-AP.htm

26) anti-FASN (Cell Signaling,3189S) can be found in 98 citations. The manufacturer also provides antibody testing data and validation:https://www.cellsignal.com/products/primary-antibodies/fatty-acid-synthase-antibody/3189

27) anti-Vinculin XP (Cell Signaling, 13901,E1E9V) can be found in 426 citations. The manufacturer provides antibody testing data and validation: https://www.cellsignal.com/products/primary-antibodies/vinculin-e1e9v-xp-rabbit-mab/13901

28) Goat Antibody Monospecific for Human Apolipoprotein (B, Beckmann Coulter,467905)

Secondary antibodies:

1) Donkey anti-rabbit IgG, conjugated to HRP antibody (GE Healthcare, NA934V). The manufacturer also provides antibody testing data: https://www.cytivalifesciences.com/en/de/shop/protein-analysis/blotting-and-detection/blotting-standards-and-reagents/amersham-ecl-hrp-conjugated-antibodies-p-06260

2) Sheep anti-mouse IgG, conjugated to HPR antibody (GE Healthcare, NA931). The manufacturer also provides antibody testing data: https://www.cytivalifesciences.com/en/us/shop/protein-analysis/blotting-and-detection/blotting-standards-and-reagents/amersham-ecl-hrp-conjugated-antibodies-p-06260

3) Donkey anti-goat lgG, conjugated to HPR antibody (Jackson Laboratories, 705-035-003) can be found in 211 citations. The manufacturer also provides antibody testing data: https://www.jacksonimmuno.com/catalog/products/705-035-003

4) Goat anti-guinea pig (Progen, 90001). The manufacturer also provides antibody testing data: https://www.progen.com/anti-guinea-pig-IgG-goat-polyclonal-HRP-conjugate/90001

5) Goat anti-mouse lgG (Vector Laboratories, BA9200) can be found in 993 citations. The manufacturer provides antibody testing data and validations : https://www.2bscientific.com/Products/Vector-Laboratories/BA-9200/Goat-Anti-Mouse-IgG-Antibody-HL-Biotinylated

6) Goat anti-rabbit lgG (Vector Laboratories, BA-1000) can be found in 3625 citations. The manufacturer provides antibody testing data and validation: https://www.2bscientific.com/Products/Vector-Laboratories/BA-1000/Goat-Anti-Rabbit-IgG-Antibody-HL-Biotinylated

7) Goat anti-rat lgG (Vector laboratories, BA-9400) can be found in 250 citations. The manufacturer provides antibody testing data and validation: https://www.2bscientific.com/Products/Vector-Laboratories/BA-9400/Goat-Anti-Rat-IgG-Antibody-HL-Biotinylated

8) Donkey Anti-Sheep IgG NorthernLights™ NL557- conjugated Antibody(bio-techne, NL010) can be found in 13 citations. The manufacturer provides antibody testing data and validation: https://www.bio-techne.com/p/secondary-antibodies/donkey-anti-sheep-igg-northernlights-nl557-conjugated-antibody_nl010

9) Goat anti-mouse Alexa 488 (Molecular Probes, A1101) can be found in 2708 citations (https://www.biocompare.com/9776-Antibodies/7011934-Goat-anti-Mouse-IgG-H-L-Secondary-Antibody-Alexa-Fluor-488-conjugate/#citations).

10) Goat anti-guinea pig Alexa 633 (Molecular Probes, A21105) can be found in 84 citations (https://www.citeab.com/antibodies/2401222-a-21105-goat-anti-guinea-pig-igg-h-l-highly-cross)

Eukaryotic cell lines

Policy information about cell lines

Cell line source(s)	IEC-6 cells (ACC 111) were purchased from the Leibniz Institute DSMZ – German collection of microorganisms and Cell Cultures GmbH (lot 5, 18.06.2015).
Authentication	The cell lines were not authenticated.
Mycoplasma contamination	The cell lines were routinely tested and confirmed negative for mycoplasma contamination.
Commonly misidentified lines (See ICLAC register)	No ISLAC cell lines were used in this study

Animals and other organisms

Policy information about studies involving animals; ARRIVE guidelines recommended for reporting animal research

Laboratory animals	The study used the following mouse lines: Dars2fl/fl (Dogan, S. A. et al., 2014), Cox10fl/fl (Diaz, F. et al. 2005), Vil1-Cre (Madison, B. B. et al., 2002) and Villin-CreERT2 (el Marjou, F. et al., 2004). Sdhatm2a mice were obtained from the Knock Out Mouse Project (KOMP) repository (Project ID: CSD48939) and bred to FLP deleter mice (Rodríguez, C. I. et al.,2000) to delete the FRT-flanked region to generate Sdhafl/fl mice.<br>Both female and male mice between 1 – 12 weeks of age were used in all in vivo experiments which is specified in figure legends for each experiment, whereas metabolic tracing studies were performed exclusively on male mice. Littermates not carrying the Vil1-Cre or Villin-CreERT2 transgenes were used as controls in all experiments. All mice were maintained in C57BL/6N background. Mice were housed at the specific-pathogen-free (SPF) animal facilities of the CECAD Research Center of the University of Cologne under a 12h dark/12h light cycle in individually ventilated cages (Greenline GM500; Tecniplast) at 22 (±2)°C and a relative humidity of 55 (±5) %. All mice had unlimited access to water and fed with a standard chow diet (Harlan diet no. 2918 or Prolab Isopro RMH3000 5P76) ad libitum. For the experiments assessing the role of dietary fat, mice were fed with fat-free diet (E15104-3474, ssniff-Spezialdiäten GmbH, Soest, Germany).<br>The study used the following Caenorhabditis elegans strains: Bristol N2, RT1315 unc-119(ed3); pwIs503[pvha-6::mans::gfp;cbr-unc-119], VS25 hjIs14 [vha-6p::GFP::C34B2.10(SP12) + unc-119(+)] and RT130 pwIs23 [vit-2::GFP]. All the experiments were performed with hermaphrodites worms at day 1 and 4 of adulthood that is indicated in the corresponding figures and/or figure legends
Wild animals	The study did not involve wild animals.
Field-collected samples	The study did not involve samples collected from the field.
Ethics oversight	All animal procedures included in the study were conducted in accordance with European, national and institutional guidelines and protocols were approved by local government authorities (Landesamt für Natur, Umwelt und Verbraucherschutz Nordrhein-Westfalen, Germany) and Animal Welfare Officers of University Medical Center Hamburg-Eppendorf and Behörde für Gesundheit und Verbraucherschutz Hamburg, Germany.<br>In this research, invertebrate C. elegans was used as an organismal model and no ethical approval was required. According to the "Zentrale Kommission für die Biologische Sicherheit" (ZKBS), the responsible entity inside the Bundesamt für Verbraucherschutz und Lebensmittelsicherheit to assess the risk of Genetically Modified Organisms (GMO), genetic work with C. elegans is classified as risk group 1 (biological safety level 1: S1). Accordingly, C. elegans work was performed in a S1-laboratory. The use of GMO in Germany is regulated by the "Gentechnik-Gesetz", and the guidelines applying to S1 work with GMO (i.e., documentation of the project and of the, exact description of the creation and maintenance of the genetic modification or correct waste treatment) were followed.

Note that full information on the approval of the study protocol must also be provided in the manuscript.
